# Research on Color Space Perceptions and Restorative Effects of Blue Space Based on Color Psychology: Examination of the Yijie District of Dujiangyan City as an Example

**DOI:** 10.3390/ijerph17093137

**Published:** 2020-04-30

**Authors:** Jiangjun Wan, Yawen Zhou, Yuxin Li, Yi Su, Ying Cao, Lingqing Zhang, Liu Ying, Wei Deng

**Affiliations:** 1 School of Architecture and Urban-Rural Planning, Sichuan Agricultural University, Du Jiangyan, Chengdu 611830, China; 201608436@stu.sicau.edu.cn (Y.Z.); liyuxin@stu.sicau.edu.cn (Y.L.); yingcao@sicau.edu.cn (Y.C.); 41360@sicau.edu.cn (L.Z.); 2 Rural Development Research Institute, Sichuan Academy of Social Science, Chengdu 610041, China; 201608383@stu.sicau.edu.cn; 3 College of Geography and Resources Science, Sichuan Normal University, Chengdu 610101, China; liuying@imde.ac.cn

**Keywords:** blue space, color space, perception and restorative evaluation, logistic regression model, semantic differential method

## Abstract

An increasing number of studies have demonstrated that blue space can promote the recovery of the human body, as does green space. However, the influence of blue space on the color space has been studied much less. Based on research on color psychology and healthy cities, the current study takes Yijie District of Dujiangyan City as the research object. Logistic regression models and the semantic differential method was used to explore residents’ perceptions of color space and their restorative ratings of blue space. This study describes the characteristics of the people visiting blue space, investigates whether the benefits brought about by blue space are related to the color space, and studies whether the surrounding colors in the blue space have a positive effect in promoting residents’ perceptions of pleasure. Based on residents’ choices of environmental pictures containing various colors, a color tendency map was constructed. The results revealed strong correlations between residents’ perceived pleasure and color space color collocation. Socio-demographic factors were found to affect the frequency of visiting blue space. Reasonable color planning is necessary for human health. Based on the study findings, relevant planning suggestions are proposed as a reference basis for waterfront space color planning.

## 1. Introduction

### 1.1. Blue Space Plays an Important Role in Urban Development

Since ancient times, water has played a primary role in human existence, through irrigation and transportation, and has carried the rise and fall of cities [1]. Blue space is no longer simply an indispensable resource. It is a crucial feature to be considered in all future developments, in order to provide humans with the opportunity to escape from their congested lives [2]. In the past 20 years, with the increasing attention on water resources in cities, the transformation and remodeling of urban blue space has gradually been put on the agenda. An increasing number of studies have begun to focus on the development of blue space from a human point of view.

In terms of the overall process, development of blue space generally follows a deterioration–suburbanization–decay–revival pattern [3]. Since 1961, the blue space in many developed countries has been subject to an anti-industrial revolution. In 1977, in Japan’s third national comprehensive development plan, shore development, blue space development, and waterside development were positioned as the basic foci of urban blue space development [4]. The combination of water and green space, building and environment, has further emphasized the importance of “water” in urban blue space [5]. Before the 21st century, with respect to urban blue space, transformation and development were mainly divided into internal reorganization methods and external expansion models [6]. However, since the beginning of the 21st century, cities no longer rely on economic and trade-based transformation plans; instead, they have begun to consider the “human-oriented” characteristics and effects of blue space.

Water is at the center of a range of environments, and has an identifiable potential to promote human health. Like green space, it has the potential to improve human comfort and promote human resilience [7]. The relationship between water and health has been at the core of early treatment and landscape research [8]. A study of the relationship between coastal blue space and health in England, which began in 2009, collected participants’ activities, motivations, and emotional attitudes in relation to blue space. The results demonstrated that access to blue space is more likely to satisfy physical and activity purposes when compared to access to other types of spaces [9]. Triguero-Mas found that access to blue spaces can increase social interaction to some extent [10]. Further, several studies from the United Kingdom and the Netherlands have shown that the use of blue space is associated with good mental health [11]. The distance between one’s living environment and the blue space may influence this association, with evidence suggesting that living more than 1 km from the blue space is associated with positive health effects, but living less than 1 km from the blue space may have negative health effects [12]. The use of urban coasts, rivers, and lakes as spaces for recreation, exercise, and recovery can ease the pressure caused by saturation of cities [13].

In general, at present, blue space has undergone commercialization, and can be considered part of an interdisciplinary research model. Based on the transformation of the original business model, more attention has been paid to satisfying residents’ environmental needs. This type of research is still in its infancy, and has not been extensively addressed in mainstream research literature. The research on this topic is obviously insufficient, but the potential of water for human promotion is indeed indisputable. There is still great potential for research in the future. Studies of waterfront space have penetrated deeply into various disciplines, including architecture and geology, and have become a hot topic of interest.

### 1.2. Reasonable Color Planning Is Necessary for Human Health

Color psychology is a branch of psychology that assumes that color has a series of psychological or behavioral responses. Color psychology, like color therapy, is not accepted by mainstream medical science, but there are still a large number of research articles discussing the psychological, cognitive, biological, and behavioral effects of color [14]. Ou categorized colors that affect psychology according to dynamics, mass, and warmth [15,16]. These three color variables were used to analyze the effects of colors on human psychological changes. The results were basically the same as those of Gao [17]. It is generally believed that the emotional connotation of color is mainly related to lightness and chroma, and less related to hue.

**Figure 1:**
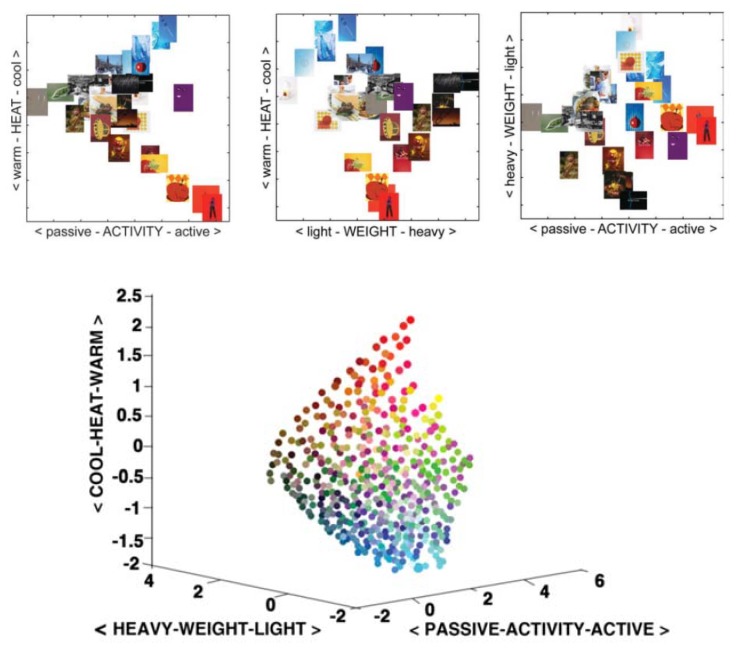
Three-dimensional emotional color space.

At present, there is little research evidence indicating that color can convey emotions; however, when inputting words such as “warm”, “cool”, and others into the retrieval system, corresponding pictures will be automatically retrieved. Based on the association between color and emotion, Solli and Lenz [18] designed a color model to discuss this relationship (Figure 1). This color model and related research results have provided a reference for color therapy and color geography, and also play a decisive role in urban color research and psychological cognition research.

Many studies have shown that there is an inherent correlation between color and geographic environment [19]. Different colors are of different wavelengths, and each vibrates to a different degree upon visual stimulation. These vibrations then visually transmit to the cerebral cortex, producing a certain stimulating effect on the whole body, and in turn affecting human health from multiple aspects, including physiology, psychology, and emotion [20]. Thus, it can be seen that color planning and design in relation to blue space are not only based on aesthetics, but also have the potential to restore and promote the physical and mental health of urban residents who engage in blue spaces to varying degrees [21].

To date, research on urban color has typically focused on historical and cultural districts and areas. Unified color planning can only unify urban color; it is still impossible to prove whether it impacts on the inner perceptions of urban residents. There is little research on therapeutic and stimulative color planning and design. In recent years, studies of urban color planning and color psychology have been on the rise. In addition to their own fields, these studies have penetrated into fields such as the architecture and color therapy fields, which reflects their great research potential.

### 1.3. Research Objectives

This research combines psychology, planning, landscape science, and environmental behavior science to examine blue space from the perspectives of different fields. At the same time, it scientifically identifies color arrangements that can improve human mood to promote the happiness of urban residents and the overall image of the city. This research will attempt to achieve three objectives: (1) demonstrate that there is a relationship between residents’ cognition and color space, (2) demonstrate that reasonable color planning is necessary for human health, and (3) propose relevant planning suggestions as a reference for blue space color planning.

## 2. Data Sources and Methods

### 2.1. Research Area

**Figure 2:**
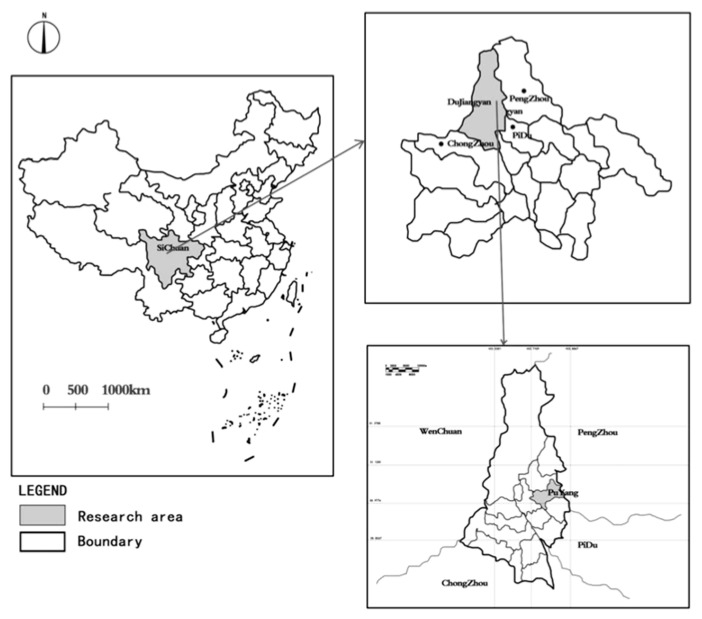
Survey area location.

For the purpose of this study, we defined waterfront space as “the terrestrial margin adjacent to the water”, which falls within the nine space types defined by Brand as waterfront squares [22]. Color space was defined as “all color elements within the waterfront and its delineated land area”. The area under study is the Yijie District of Dujiangyan city, Chengdu city, Sichuan province, located in the south of Puyang town, in the north district of Dujiangyan city. The study area covers an area of about 1.5 square kilometers. There are 690 m of built landscape river and 66 acres of artificial lake, known as Yi Lake. There is a public sports and leisure park of 60 mu with a green rate of 36.6%; this is a standard urban blue space, and as such, it is the target of this research (Figure 2).

**Figure 3:**
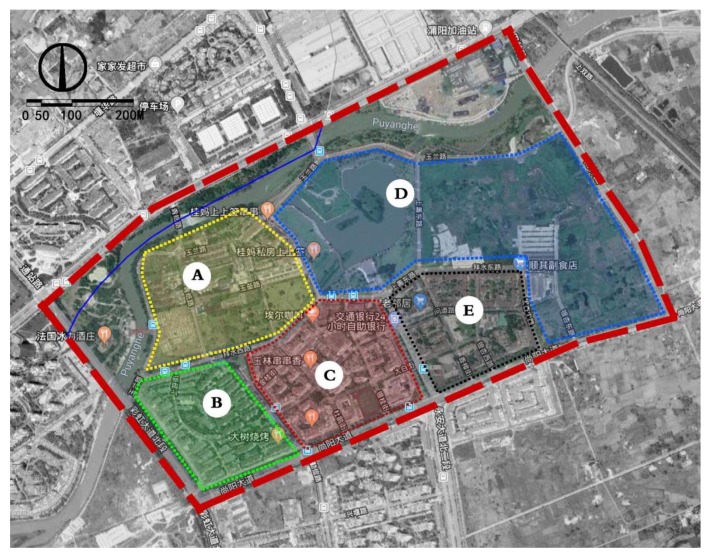
Research area division.

This research is based on the overall planning of Yijie District of Dujiangyan. The two main roads on this block, the south and west road of Shangshan and the east and west road of Baishui, were taken as the boundaries. The areas enclosed by the main roads, and water body was divided into five typical small-range study areas—A, B, C, D, and E—for map sampling (Figure 3). Area A is a moderate distance from the water body, with white buildings and abandoned walls in the west in addition to conventional greenery; there are newly repaired yellow buildings in the middle and east. Area B is the farthest from the water body, with a mixture of white and yellow buildings. Area C is a moderate distance from the water body, with brownish-yellow renovated buildings together with a mix of some old white buildings. Area D is at the center of the water body, with open green space and partly abandoned buildings; no buildings in this area are in use. Area E is a moderate distance from the water body, with more than 90% of the buildings built in recent years; the buildings are mostly brownish-yellow in color.

The data collection methods included questionnaires, observations, and interviews. The questionnaire investigated residents’ or tourists’ perceptions of and preferences for color space in the blue space of Yijie District, the observation method involved the observation and recoding of the color collocation of buildings, landscapes, environmental waters, and other areas in the study area. The interview method was used to understand the needs and satisfaction of residents and tourists with respect to the environmental elements in the waterfront space. Because the geographical locations of different subdivisions are various distances from the central water body, zoning investigations were carried out according to the divisions depicted in the below figure.

### 2.2. Research Methods

The purpose of this research was to examine the correlation between residents’ perceived pleasure and color space collocation, and to investigate the need for reasonable color planning for human health. SPSS 25 (SPSS Inc., Chicago, IL, USA) statistical software was used to test the reliability and validity of the questionnaire, in order to verify its scientific properties and preciseness. Logistic regression models were used to explore the key factors influencing the frequency that residents visited blue space. The semantic differential (SD) method and factor analysis method were used to evaluate residents’ perceptions from various angles.

Different analysis methods were adopted for different data: (1) multiple reliability and validity tests were conducted for all questionnaire items, and multiple pre-surveys were conducted after the exclusion of redundant items; (2) given that the adjectives and adverbs used to describe residents’ overall perceptions of color space and blue space in the questionnaire were divided into five levels, which met the scoring requirements of the SD method, the SD method was used to analyze the data; (3) in terms of the relationships between the frequency of visiting blue space and relevant factors, logistic regression was used to conduct one-to-many significance analysis and to obtain significance values; (4) finally, the color tendency analysis data for blue space and color space were obtained through the questionnaire, and one-sample *t*-tests were performed to obtain the tendency results of different groups for different colors. The above analyses were completed using SPSS 25 software.

## 3. Research Design and Process

### 3.1. Variable Design

See Table 1 for an overview of the variables. Considering the potential influence of various personal factors on residents’ perceptions, several personal variables were measured, including gender, nationality, education level, occupation, duration of residence in the region, visual health, and frequency of visiting blue space (Table 1). In addition, we also set up multi-factor variables, such as cognitive choice and color tendency for reference.

### 3.2. Questionnaire Design

The questionnaire design was based on five dimensions: architecture, nature, landscape, city, and cognitive color. The questionnaire was divided into three parts: basic information (personal information, including age, gender, occupation, etc.), preference test (selection of pictures with difference scenes), and tendency test (selection of different colors in the scene pictures). The preference test involved 42 variables. Participants were asked to select as many adjectives with opposite meanings as possible in relation to six evaluation levels: aesthetics, relaxation, psychology, physiology, vision, and collocation. Five responses were then set between each pair of adjectives, and the respondent was to choose the response that best fit their perception: “very like”, “like”, “general”, “dislike”, and “very dislike” were used to distinguish participants’ preferences and tendencies for color. The values −2, −1, 0, 1, and 2 were assigned from left to right, respectively, for numerical processing in quantitative analysis. This was also used as the basis for obtaining the semantic difference scale (i.e., the SD curve).

### 3.3. Pre-Investigation and Questionnaire Revision

This research covered the whole Yijie District of Dujiangyan city. The sample was a random probability sample recruited from activity groups who entered the target area during the specified time periods, i.e., 9:00–11:00 a.m., 12:00–2:00 p.m., 4:00–6:00 p.m., and 8:00–10:00 p.m. These four sample periods were denoted as morning, noon, afternoon, evening; each sample period went for two hours.

Trained interviewers administered questionnaires randomly to all residents visiting the blue space in one block, and interviewed some of these residents. They only looked for participants in selected areas, at least 2–3 times, during the four periods denoted above, in order to obtain data at different time points. During the investigation, the survey rate was usually between 85% and 90%. This investigation began on 15 September 2019; two pre-investigations were conducted between 15 September and 30 September 2019.

We tested the reliability of the results obtained from two pre-investigations in sequence. The first investigation of the reliability of the valid questionnaire returned a reliability of 0.56. Based on this, two inefficient indicators were removed for the second pre-investigation. The reliability of the second valid questionnaire was 0.64. A low-efficiency index was removed, and the final formal questionnaire was finally established. The evaluation factor table is provided below (Table 2).

### 3.4. Formal Investigation

The formal survey was conducted from 4 October to 28 October 2019. A total of 500 questionnaires were issued, 100 for each region, and a total of 436 were returned; the return rate was 87.2%. In total, 427 of these were valid questionnaires, accounting for 85.4% of the distributed questionnaires.

In terms of sampling structure, we first selected merchants, who accounted for 50% of the total research group (including shopkeepers, clerks, and randomly selected consumer groups performing business transactions in stores). Secondly, we selected residents in residential areas (including people of all ages in the household, service staff in the community, etc.), who accounted for 35% of the total research group. Third, we selected activity groups in the landscape area, accounting for 10% of the total research group. Finally, we selected government officials in the area, accounting for 5% of the total research group. Considering that some of the respondents were unlikely to respond to the questionnaire, we expanded the selection probability and scale to make the data more representative.

## 4. Results

### 4.1. Quantitative Analysis of Color Space Evaluation Quality

In order to carry out targeted and in-depth research, we defined an expected color palette as a “pleasant, attractive combination”. This definition was used as the basis to further subdivide the five dimensions of architecture, nature, landscape, city, and cognitive color. The first factor was composed of the degree to which the landmark building was a representative color, the degree of coordination between the environment and architecture, the degree of color division in the block, and the degree of color coordination among key buildings and general buildings. this factor was denoted as building color (F1). The second factor was the landscape color (F2), composed of warm and cold tones and comfort level of the landscape. The third factor was natural color (F3), comprised of color matching of the water body, greenery, and landscape. The fourth factor was urban color (F4), comprised of the degree of contrast, degree of coherence, and degree of pleasure of the color. The fifth factor was comprised of perceived satisfaction, happiness, atmosphere, attractiveness, enthusiasm, and relaxation, and was denoted as cognitive color (F5). The data distribution of the five factors can be used for further research and analysis.

#### 4.1.1. Reliability Test

Reliability refers to the degree of consistency and stability of the data obtained during repeated tests of the questionnaire. Reliability estimates range from 0~1; the larger the value, the higher the reliability. Cronbach alpha (referred to as α) is used to test reliability. The mathematical formula for the Cronbach alpha coefficient is $\alpha =\frac{K}{K-1}\left(1-\frac{\sum_{i=1}^{k}\sigma_{i}^{2}}{\sigma_{T}^{2}}\right)$. Here, *K* represents the total number of items, *σ_i_^2^* represents the variance in the questionnaire score, and *σ_T_^2^* represents the variance in the total score. In SPSS, this test was carried out as follows: Analyze–Scale–Reliability Analysis, select 24 variables to be analyzed under "Item, and analyze the data. The output showed that the alpha reliability coefficient was 0.784. After deleting an inefficient index, the reliability factor was increased to 0.802. Table 3 shows the questionnaire indices and the reliability test results.

#### 4.1.2. Validity Test

Validity refers to whether a selected item can represent the measured subject; it reflects the accuracy of the measurement result. The validity of the questionnaire is more important than the reliability. After reliability analysis and the deletion of inefficient items, the items were analyzed for validity. The KMO (Kaiser-Meyer-Olkin) index was 0.903 (Table 4), which is very close to 1, indicating that the data were very suitable for factor analysis.

Factors were selected based on eigenvalues greater than 1, as proposed by Cliff [23]. Specifically, after each factor analysis, the variables with a factor loading above 0.6 were retained, and the reliability test and factor analysis were performed again. At the completion of this analysis, 24 variables were retained for further analysis (Table 5). It can be seen that the adjusted and verified questionnaire is more scientific and rigorous, and thus, is more suitable for in-depth analysis and research.

In Table 5, after excluding items (based on Table 3), the remaining items are arranged in descending order of initial eigenvalues. We can see that item 1 (color is representative of the style of the district) explained 27.981% of variance, with an eigenvalue of 6.715. There were four eigenvalues greater than 1. Item 2 (harmony of color in the architecture and environment) explained 23.494% of variance, while items 3 (warm colors feel comfortable) and 4 (water improves spatial perceptions) explained 20.284% and 9.240% of variance, respectively.

The cumulative contribution rate of the first four factors was 81%, so the validity was high. Among them, the building color factor (F1) had the largest contribution, explaining 84.848% of variance, while the second factor, natural color (F3), explained 4.326% of variance, and the third, fourth, and fifth factors, denoting cognitive color (F5), landscape color (F2), and urban color (F4), respectively, explained 2.975%, 1.632%, and 1.197% of variance.

### 4.2. Overall Perception of Blue Color Space

**Figure 4:**
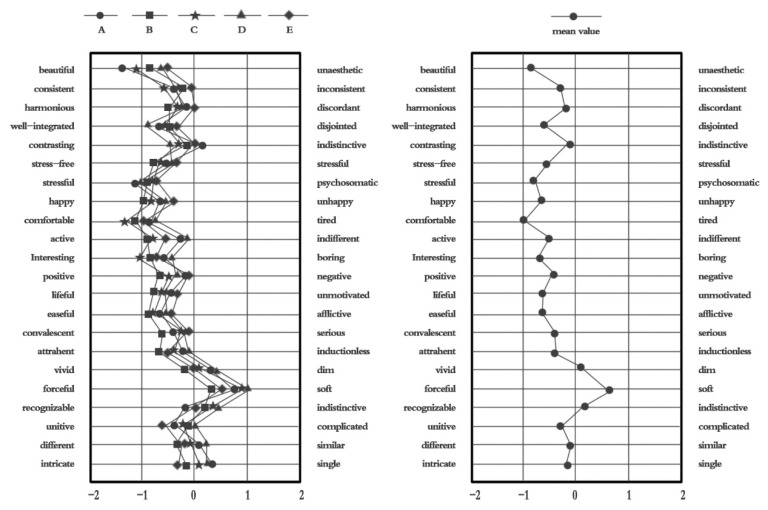
SD curve of the evaluation of blue space in the Yijie District.

Descriptive statistics for the sample data were generated, and the average score for each adjective and adverb was obtained. Based on these results, an SD curve of the evaluation of blue space in the One Street District was drawn (Figure 4). These scores are derived from psychology and are called mental quantities. The grid graph on the left represents 22 groups of mental quantities in five sample areas; the grid graph on the right represents the average mental quantities in the five sample areas.

According to the residents’ cognitive results, the D area located in the center of the landscape water body in the Yijie District tends to the left of the words “integration” of architecture and nature, and scores higher than other areas, indicating that the area near the water has better color matching and fusion. In the general residential areas C and E, most of the residents interviewed were middle-aged males and females. For various reasons, the overall free time of these residents is less than the rest of the sample, and their sense of happiness is lower. Therefore, it is generally believed that the water body has a certain effect on eliminating fatigue. Area A, where public service facilities are concentrated, has a number of iconic buildings as well as public service buildings, and the architectural landscape caters to the surrounding environment. The residents in this area have the highest aesthetics and coordination indices from among the overall survey area. The overall blue color selection and landscape categorical evaluation are moderate, and in the SD curve, the average score of the five areas was located near the “0” value.

Among the five districts, respondents generally believed that all blocks have a good degree of integration between architecture and natural colors: the aesthetics are well coordinated, the overall tone is soft, and the buildings represent the style of this area. The indicators for the psychological, relaxation, and physiological factors were to the left, indicating that with a reasonable color mix in the blue space, respondents tend to feel joy and happiness.

### 4.3. The Frequency of Visiting Blue Space Is Related to Personal Variables

In this study, 1–4 visits to the blue space daily or weekly was considered a higher frequency of visits, while 1–4 visits to the blue space in two weeks or a month was considered to be occasional visiting. As such, 35.6% of residents had a high blue space visitation frequency (*n* = 152), 59.3% of the residents had a low visitation frequency (*n* = 253), and 0.51% of the residents had never visited the blue space (*n* = 22). Because the dependent variable, visit frequency, was an ordered variable, and the independent variables were categorical variables, an ordered multi-class logistic regression model was used to explore the relationships between the frequency of visiting blue space and personal variables (take the delineated area D as the core blue space; “visit the blue space” is subject to visit D).

People who had never or occasionally visited the blue space differed in terms of gender, ethnicity, education level, occupation status, length of stay, and visual health. Among these variables, there were significant differences in education level, occupation status, and length of residence; the social profiles of the two groups are shown in Table 6. Of note, 50% (weighted of total) had a high school education, and 86.4% (weighted of total) of the non-permanent workers had never been to the blue space. Of those who occasionally visited the blue space, 91.3% (weighted of occasionally visited people) had permanent jobs, and 47.1% (weighted of occasionally visited people) were permanent residents of the district (>10 years). In addition, compared with the occasionally visiting group, those who visited more frequently had higher education levels (master’s and doctoral degrees).

In terms of the overall number of visitors, more Han people (61.7%) occasionally visited waterfront spaces compared to ethnic minorities (22.2%), but among those who frequently visited waterfront spaces, there was a greater proportion of ethnic minorities (74.1%) than Han people (33.1%). More people with permanent jobs (91.3%) occasionally visited the blue space, compared to those without permanent jobs (8.7%).

In the ordered logistic regression model, a *p* value less than 0.05 was considered statistically significant. As can be seen from Table 7, education level, residence duration, and occupation were significantly (marked with *) associated with visiting blue space.

### 4.4. Trend Results for the Color Analysis of Blue Space

In order to conduct independent analysis of the differences in perceptions of residents after visiting blue space, we used the “occasional” and “frequent” responses to the visiting frequency measure to define the groups. The 12 independent measures of tendency (Table 8) were the test variables. Then, a series of single-sample *t*-tests were performed. The process was therefore (1) analyze, (2) compare means, (3) one-sample *t*-test.

The *t*-test companion probability *p*-values of the 12 items were all less than the significance level of 0.05. Thus, for all items, there was a significant difference between the high-visitation and low-visitation groups, indicating that each single item has a certain discriminative power. This demonstrates that the questionnaire responses tended to differ significantly.

Most respondents thought that the water body in the space has a positive effect on improving comfort (70.1%). At the same time, reasonable color matching (67.1%) and coherent color connections (68.2%) were also thought to promote physical and mental happiness.

**Figure 5:**
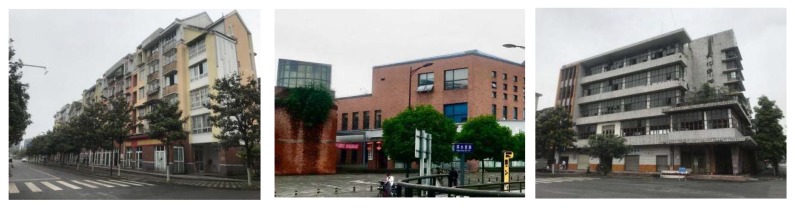
Residential building color tendency.

**Figure 6:**
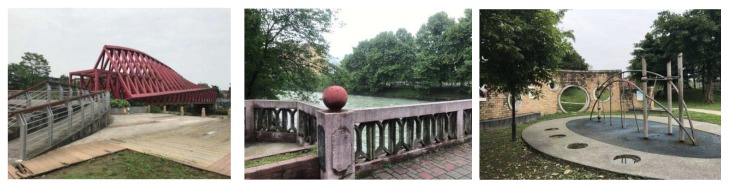
Landscape color tendency.

**Figure 7:**
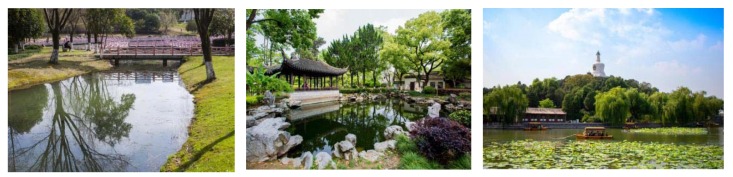
Network landscape trend color map.

**Figure 8:**
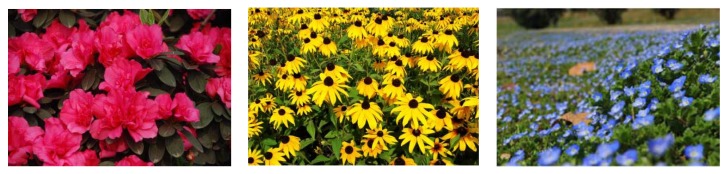
Landscape configuration orientation color map.

During the questionnaire, we presented the respondents with multiple color pictures, including pictures of the study area and other online pictures. Respondents had to choose their desired pictures according to their self-inclination. In the selection of residential buildings, 62.8% of people chose residential buildings with obvious color contrast (Figure 5). Furthermore, 83.6% of people chose rest space with water and one or two prominent color combinations (73.2%) (Figure 6 and Figure 7). Respondents preferred benches in warm colors, such as brown and earthen yellow (64.1%) compared to black or gray benches, and they also preferred flowers of bright colors, such as red and yellow (74.1%) (Figure 8).

## 5. Discussion

### 5.1. Overall Perceived Benefit of Access to Waterfront Space Is Great

In the SD psychological curve, respondents’ overall cognition in relation to visiting blue space was generally inclined to the left of the graph. This indicates that respondents were more likely to believe that visiting blue space is relaxing and pleasant, which is conducive to physical and mental health (see Figure 4).

We analyzed the scores of respondents from five sub-regions that were different distances from the waterfront space (zone D). The curves in each area were basically the same. Most respondents (73.5%) said that although it takes time to get to the blue space, as it is some distance from their residence, the distance does not influence the purpose and motivation of respondents to “relax” and “eliminate fatigue” around the blue space. When we asked respondents to score the integration of buildings and nature in blue space and general areas, area D scored higher than other areas for many indicators. This indicates strong natural integration, with the water element playing a crucial role. The water environment is obviously the first choice for relaxation and leisure.

People can have different perceptual experiences of nature and water environments, and for 90% of people, the perceptions are beneficial to the human body. Ulrich [24] compared the reactions of people to a natural landscape with water versus one with no water, and found that the water environment has a restorative effect on physical and mental health. This is clearly reflected in the current data analysis. In the current study, respondents who “occasionally” or “often” visited blue space were more likely to believe that the water environment produces obvious perceptual experiences, such as “pressure dissipation”, “restoring energy”, and “eliminating weariness”; most people think that a spatial place with a water environment is a positive and interesting atmosphere.

### 5.2. Basic Personal Conditions Significantly Affect the Frequency of Visiting Blue Space

Most of the respondents visited blue space at least once or twice every two weeks during their activity time, but the frequency of visiting blue space was related to a combination of factors. Education level, occupation, and duration of residence were all predictors of the frequency of visiting blue space.

Our research results show that socioeconomic status is one of the main factors that influences how often a resident visits blue space, and also determines how much benefit is gained from visiting blue space. Variation in socioeconomic status is associated with variation in binding forces, and as such, these waterfront areas may mitigate the health impacts of socioeconomic inequality [25]. It is worth noting that varying social and economic status brings about different social pressures. The need for blue space for recovery is also different, demonstrating that the water environment may ease the impact of socioeconomic inequality on health [26].

Those of a higher social and economic status tend to visit blue space more frequently, and most believe that they feel “relaxed” and are “enjoying the joy of nature” in blue space; this is directly reflected in their higher education level and stationary profession condition. Due to the support of social factors, these people have greater capabilities and opportunities to “appreciate the beauty of nature”. For those with a low social status or lower economic level, visiting blue space may not bring many benefits. Some of these people have lived in the research area for more than five years without having visited the blue space. This appears to be related to low levels of education and occupational instability; these people experience life pressures greater than the energy they receive from nature. They may also have less opportunity to visit blue space, and thus, cannot obtain benefit from the water environment. In addition, blue space may be viewed negatively be these people, as an unhealthy place [27]. It has also been shown that the benefits people gain from natural space are the result of the interaction between individuals and the wider social environment [28].

Furthermore, the results revealed that older respondents and those who had lived in the area for a long time were more likely to visit the blue space as one of their basic activities. It was interesting to find that more than 90% of the people who visited the research area for the first time chose to visit the target water environment because of its tourist and leisure attraction. For these respondents, visiting blue space with a recreational pursuit can bring benefits [29].

### 5.3. Residents Have an Obvious Color Tendency When They Visit Blue Space

The results show that color is an important factor affecting the quality of visiting perceptions. In total, 67.1% of respondents thought that reasonable color collocation can promote physical and mental well-being. This result is reflected in their selection of relevant pictures. More interviewees tended to choose warm color environments with bright colors, believing that “1–2 prominent colors” in the environment is the most appropriate color combination.

At the same time, we found that certain colors were considered appropriate for certain facilities in the space, such as access paths and benches; these facilities are key to obtaining benefits from blue space [27]. Most respondents preferred “red” and “yellow” flowers to “white” and “blue” flowers; most preferred “warm coffee color” and “brick red” to “gray” and “black” benches; most preferred “coffee color” wooden corridors to “gray black” concrete roads. This also shows that color collocation around a water space can have greater effects on people. This may be because different colors bring different benefits. We combine this with the definition of psychological colors in color psychology: cold colors mostly symbolize calmness and solemnity, but also indifference and defense, while warm colors mostly symbolize enthusiasm and cheerfulness, which can soften attacks; thus, we argue that “warm colors” are more positive and have more resilience than “cool colors”.

It is worth noting that from the images that were presented to participants, 80% of respondents chose images that showed a picture of water. This indicates that these water environments are even more attractive to respondents, indicating that humans have a natural affinity for water environments [30]. The analysis results we obtained also tended to coincide with the actual survey responses. When conducting the sample interviews, we noted that a number of interviewees gave similar responses:

“I’ve been retired for a long time and enjoy coming here to be active, where I can see water, trees and all kinds of flowers and plants, and lots of people my age, and everyone is a little happier together”

(from a 67-year-old woman).

“I like to take my kids to play on this side of the Red Bridge, sometimes the weather is bad and it’s grey everywhere I go, but it’s always brightly colored and the kids choose this place every time”

(from a woman who has a son).

“I’m busy at work and don’t get many opportunities to come out and relax, but when I’m free, I’ll still have tea by the river and be in a calmer mood”

(from a middle-aged man).

From an evolutionary perspective, the attraction to water and color makes sense. Early humans attracted to freshwater were more likely to survive than those attracted to non-aquatic environments [31]. These primitive natural selection factors further suggest that we have evolved a preference for water environments [32].

## 6. Conclusions

Blue space is an important type of living space, and has great importance for the emotional and mental health of residents. In our research, most people “occasionally” or “often” visited blue space, and almost all of these visitors had high levels of education and stable occupations. Therefore, the frequency of visiting blue space is determined by the social and economic status of the person. Blue space has strong natural attributes and plays a crucial role in emotion perception and psychological recovery. For disadvantaged people in society, the benefits of water environments cannot be accessed due to individual limitations. Most people believe that color choices in blue space are significant in creating a water environment atmosphere. Positive color collocation is conducive to eliminating negative impacts brought about by social pressures, while colors that contrast with natural colors are associated with increased visitation, due to increased attractiveness and visual stimulation. This suggests that the quality of colors in a blue space may be the basis for the relaxation effects of these spaces. However, there are also some limitations of this research. For example, we did not conduct a comparative study of people of different ages. Samples were also not collected from other cities in order to make comparisons between cities. In the future, researchers should focus on the above two key points. At the same time, city planners and managers should pay more attention to the planning of blue space, especially in relation to collocation and design of color. Future studies are needed to study different color collocations, in order to promote residents’ emotional and mental health through reasonable blue space color collocation.

## Figures and Tables

**Table 1:** Basic overview of the sample (*n* = 427).

Items	*n*	Weighted (%)
Gender		
Male	274	64.2
Female	153	35.8
Nationality		
minority	27	6.3
Han	400	93.7
Level of higher education		
Below high school	249	58.3
High school	101	23.7
Junior college and above	77	18.0
Permanent job		
Yes	352	82.4
No	74	17.6
Visit frequency		
Frequently	152	35.6
Occasionally	253	59.3
Never	22	5.1
Duration of residency		
1–5 years	104	24.4
5–10 years	64	15.0
10 years and above/visitor	259	60.7
Vision health		
Yes	425	0.98
No	2	0.02

**Table 2:** Color space perceptions and recovery evaluations of waterfront semantic differential (SD) factor.

No.	Dimensionality	Main Purpose	Evaluation Factor
1	Aesthetics	The degree of harmonious aesthetic of the color space in the waterfront space	beautiful–unaesthetic
2			consistent–inconsistent
3			harmonious–discordant
4			well-integrated–disjointed
5			contrasting–indistinctive
6	Relaxation	The comfort of residents in the color space and their happiness	stress-free–stressful
7			stressful–psychosomatic
8			happy–unhappy
9			comfortable–tired
10	Psychology	The color space creates space quality and a positive orientation	active–indifferent
11			interesting–boring
12			positive–negative
13	Physiology	The residents of the color environment achieve physical recovery	lifeful–unmotivated
14			easeful–afflictive
15			convalescent–serious
16	Vision	The degree of association between residents’ visual perceptions and color collocation	attrahent–inductionless
17			vivid–dim
18			soft–forceful
19	Collocation	The degree of color unity in the blue space	recognizable–indistinctive
20			unitive–complicated
21			different–similar
22			intricate–single

**Table 3:** Questionnaire indices and reliability test list.

Dimension	Indices	Cronbach’s Alpha if Item Deleted	Adjusted Indices	Cronbach’s Alpha if Item Deleted
Architectural color	Landmark buildings are representative (PH1)	0.765	Landmark buildings are representative (PH1)	0.792
	Markers are in harmony with the surrounding environment (PH2)	0.768	Markers are in harmony with the surrounding environment (PH2)	0.796
	Residential buildings harmonize with the environment (PH3)	0.766	Residential buildings harmonize with the environment (PH3)	0.793
	The color of the library harmonizes with the environment (PH4)	0.764	The color of the library harmonizes with the environment (PH4)	0.819
	The most comfortable architectural color (PH5)	0.763	The most comfortable architectural color (PH5)	0.791
	Distinct color division (PH6)	0.784	Distinct color division (PH6)	0.785
Landscape color	Warm colors make people comfortable (PH7)	0.756	Warm colors make people comfortable (PH7)	0.793
	The most comfortable landscape color (PH8)	0.765	The most comfortable landscape color (PH8)	0.800
	The most comfortable park (PH9)	0.772	The most comfortable park (PH9)	0.808
Natural color	Water comfort (PH10)	0.783	Water comfort (PH10)	0.809
	Color type (PH11)	0.781	Color type (PH11)	0.808
	Color transition nature (PH12)	0.762	Color transition nature (PH12)	0.813
	Complex color matching (PH13)	0.773	Complex color matching (PH13)	0.797
	Field path arrangement (PH14)	0.782	Field path arrangement (PH14)	0.809
	Flowers set (PH15)	0.774	Flowers set (PH15)	0.785
	Rest seat arrangement (PH16)	0.765	Rest seat arrangement (PH16)	0.785
Urban color	Strong collocation contrast (PH17)	0.755	Strong collocation contrast (PH17)	0.803
	Coherent color matching (PH18)	0.777	Coherent color matching (PH18)	0.802
	Whether the match is pleasant (PH19)	0.777	Whether the match is pleasant (PH19)	0.799
Cognitive color	Be satisfied with the color environment (PH20)	0.773	Be satisfied with the color environment (PH20)	0.802
	Increase happiness (PH21)	0.776	Increase happiness (PH21)	0.824
	You relax outdoors (PH22)	0.810	Create a pleasant atmosphere (PH22)	0.824
	Create a pleasant atmosphere (PH23)	0.790	Be attractive (PH23)	0.816
	Be attractive (PH24)	0.802	Positive guiding force (PH24)	0.815
	Positive guiding force (PH25)	0.793		

**Table 4:** KMO and Bartlett’s test.

KMO	Bartlett’s Test of Sphericity		
	Approx. Chi-Square	Df	Sig
0.903	12416.302	276	0.000

Note: KMO: Kaiser-Meyer-Olkin; Df: Degree of Freedom; Sig: Significance.

**Table 5:** Explanation of total variance.

No.	Initial Eigenvalues			Extraction Sums of Squared Loadings			Rotation Sums of Squared Loadings		
	Total	% of Variance	Cumulative %	Total	% of Variance	Cumulative %	Total	% of Variance	Cumulative %
1	6.715	27.981	27.981	6.715	27.981	27.981	5.909	24.620	24.620
2	5.638	23.494	51.474	5.638	23.494	51.474	5.647	23.530	48.150
3	4.868	20.284	71.758	4.868	20.284	71.758	4.139	17.246	65.396
4	2.218	9.240	80.998	2.218	9.240	80.998	3.744	15.602	80.998
5	0.924	3.850	84.848						
6	0.818	3.408	88.256						
7	0.362	1.508	89.763						
8	0.317	1.322	91.085						
9	0.254	1.058	92.143						
10	0.203	0.847	92.989						
11	0.190	0.791	93.780						
12	0.185	0.772	94.553						
13	0.173	0.722	95.275						
14	0.149	0.622	95.896						
15	0.138	0.573	96.469						
16	0.133	0.555	97.025						
17	0.121	0.505	97.530						
18	0.108	0.451	97.980						
19	0.103	0.428	98.409						
20	0.089	0.370	98.779						
21	0.081	0.337	99.116						
22	0.078	0.326	99.442						
23	0.073	0.305	99.747						
24	0.061	0.253	100.000						

**Table 6:** Social profile of those who frequently visited the blue space (*n* = 152), those who had never visited the blue space (*n* = 22), and those who visited occasionally (*n* = 253).

Items	Never			Occasionally		
	*n*	Weighted %	Weighted of Total%	*n*	Weighted %	Weighted of Total %
Gender						
Male	12	54.5	4.3	124	49	45.2
Female	10	45.5	6.5	129	51	84.3
Nationality						
minority	1	4.5	3.7	6	2.3	22.2
Han	21	95.5	5.2	247	97.7	61.7
Level of Education						
Below High School	7	31.8	2.8	123	48.6	49.6
High school	11	50	10.7	79	31.2	76.7
Junior college and above	4	18.2	5.3	51	20.2	67.1
Permanent Job						
Yes	3	13.6	0.85	231	91.3	65.6
No	19	86.4	25.6	22	8.7	29.7
Living Time						
1 year and below	7	31.8	6.6	83	32.8	78.3
1–2 years	2	9.1	3.2	26	10.3	41.9
2–5 years	2	9.1	3.6	13	5.1	23.2
5–10 years	3	13.6	4.1	12	4.7	16.7
10 years and above	8	36.4	6.1	119	47.1	90.8
Vision Health						
Yes	21	95.4	4.9	235	100	55.3
No	1	4.6	50	-	-	-

**Table 7:** Estimated parameters in ordered logistic model.

Variable	Items	Std. Error	Df.	*p*	95% Confidence Interval	
					Lower Bound	Upper Bound
**DV (Dependent Variable)**	Never	0.580	1	0.058	−2.031	0.035
	Occasionally	0.526	1	0.000	1.307	3.446
	Frequently	0.535	1	0.000	−1.015	0.026
**IV (Independent Variable)**	Male	0.207	1	0.648	−1.035	−0.220
	Female	-	0	-	-	-
	Minority	0.403	1	0.432	−0.473	1.106
	Han	-	0	-	-	-
	Below high school *	0.272	1	0.000	−1.098	−0.034
	High school *	0.311	1	0.000	−0.957	0.255
	Junior college and above	-	0	-	-	-
	Have permanent job *	0.265	1	0.028	−0.798	−0.232
	No permanent job	-	0	-	-	-

* *p* < 0.05.

**Table 8:** One-sample *t*-test.

Items	*t*-Test for Equality of Means				HL	*n*	Mean	Std. Deviation
	*t*	Df.	*p*	MD				
Color is representative of the style of the districts	4.829	403	0.000	0.32	1	253	0.95	0.542
					2	152	0.63	0.800
Harmony of color in the architecture and environment	4.296	403	0.000	0.26	1	253	1.1	0.662
					2	152	0.84	0.435
Warm colors feel comfortable	5.734	403	0.000	0.40	1	253	1.06	0.626
					2	152	0.66	0.779
Water improves spatial perceptions	4.752	403	0.000	0.31	1	253	1.24	0.466
					2	152	0.94	0.838
Color transitions smoothly from nature	5.630	403	0.000	0.22	1	253	1.23	0.447
					2	152	1.01	0.242
Complex color matching	6.029	403	0.000	0.39	1	253	1.04	0.683
					2	152	0.66	0.529
Reasonable colors can promote the happiness index	4.594	403	0.000	0.12	1	253	1.15	0.37
					2	152	1.18	0.43
Strong color contrast	5.661	403	0.000	0.41	1	253	1.07	0.659
					2	152	0.66	0.777
Good colors can feel cheerful	4.539	401	0.000	0.23	1	253	1.58	0.51
					2	152	1.62	0.777
Will choose the blue space to relax	4.817	403	0.000	0.25	1	253	1.55	0.613
					2	152	1.6	0.576
Environmental colors are attractive	−6.625	403	0.000	−0.42	1	253	0.75	0.698
					2	152	1.16	0.45
Environmental colors have positive guiding power	3.984	403	0.000	0.17	1	253	0.83	0.378
					2	152	0.66	0.49

Note: MD: Mean Difference; HL: High & Low.
